# Subgroup analyses from patients with pre-treated metastatic colorectal cancer receiving trifluridine/tipiracil: results of the TALLISUR trial

**DOI:** 10.1186/s12885-024-12599-7

**Published:** 2024-07-23

**Authors:** Meinolf Karthaus, Volker Heinemann, Jorge Riera-Knorrenschild, Albrecht Kretzschmar, Manfred Welslau, Ulrich Kaiser, Henning Pelz, Thomas J. Ettrich, Swantje Held, Linde Kehmann, Jürgen Hess, Timo Reisländer, Lena Weiss, on behalf of the TALLISUR study group

**Affiliations:** 1 Clinic for Haematology and Oncology, Klinikum Neuperlach, Oskar-Maria-Graf-Ring 51, 81737 Munich, Germany; 2 Medizinische Klinik Und Poliklinik III, Klinikum Der Universität München, Marchioninistr. 15, 81377 Munich, Germany; 3 Universitätsklinikum, Baldingerstr. 1, 35043 Marburg, Germany; 4 MVZ Mitte Leipzig, Johannisplatz 1, 04103 Leipzig, Germany; 5 Praxis Aschaffenburg, Elisenstr. 26, 63739 Aschaffenburg, Germany; 6 ÜBAG MVZ Dr. Vehling-Kaiser GmbH, Achdorfer Weg 5, 84036 Landshut, Germany; 7 Ambulantes Therapiezentrum Für Hämatologie Und Onkologie, Ebertplatz 12, 77654 Offenburg, Germany; 8 Department of Internal Medicine I, Ulm University Hospital, Albert-Einstein-Allee 23, 89081 Ulm, Germany; 9 ClinAssess GmbH, Abteilung Biometrie, Werkstättenstr. 39B, 51379 Leverkusen, Germany; 10 Medical Affairs, SERVIER Deutschland GmbH, Elsenheimerstr. 53, 80687 Munich, Germany

## Abstract

**Background:**

In the pivotal phase III RECOURSE trial, trifluridine/tipiracil (FTD/TPI) improved progression-free and overall survival (PFS, OS) of patients with pre-treated metastatic colorectal cancer (mCRC). Subsequently, the TALLISUR trial provided post-authorisation efficacy and safety data and patient-reported outcomes on quality of life (QoL) in a German patient cohort. The present analysis reports the final data on efficacy, safety and QoL and investigates the impact of baseline characteristics and associated prognostic subgroups on outcome.

**Methods:**

In this prospective, multi-centre, Germany-wide, phase IV study, patients with pre-treated mCRC were given the choice to receive either FTD/TPI or best supportive care (BSC). To assess the primary endpoint, QoL, EORTC QLQ-C30 questionnaires were employed. Secondary endpoints included QoL assessed through EQ-5D-5L questionnaires, OS, PFS and safety. Additionally, 3 subgroups were defined according to a post-hoc analysis of the RECOURSE trial: best, good and poor prognostic characteristics (BPC, GPC, PPC). Patients with < 3 metastatic sites at inclusion and/or ≥ 18 months from diagnosis to inclusion were considered to have GPC. GPC patients without liver metastasis at inclusion were considered to have BPC. All remaining patients were considered to have PPC.

**Results:**

Of 195 patients, 186 decided to receive FTD/TPI and 9 to receive BSC. The low number of patients in the BSC-arm did not allow statistically meaningful analyses. Treatment with FTD/TPI was associated with maintained QoL. For all patients, median OS was 6.9 months (95% CI 6.1 – 8.3) and for the defined subgroups (BPC *n* = 20 vs GPC *n* = 65 vs PPC *n* = 121) 12.2, 7.9 and 6.8 months (95% CI 6.0 – 18.2, 6.2 – 13.3, 5.4 – 8.1). The most frequent TEAEs were neutropenia (29.6%), anaemia (24.7%) and nausea (23.7%). Febrile neutropenia occurred in 1.1%.

**Conclusions:**

Treatment of patients suffering from pre-treated mCRC with FTD/TPI was associated not only with prolonged survival and delayed progression, but also with maintained QoL. Independent of other baseline characteristics such as ECOG performance status and age, low metastatic burden and indolent disease were factors associated with favourable outcome.

**Clinical trial registration:**

EudraCT-Number 2017–000292-83, first registration 19/06/2017.

**Supplementary Information:**

The online version contains supplementary material available at 10.1186/s12885-024-12599-7.

## Background

Trifluridine/tipiracil (FTD/TPI) is approved by the European Medicines Agency as monotherapy for the treatment of adult patients with mCRC who have been previously treated with or are not considered candidates for available therapies including fluoropyrimidine-, oxaliplatin- and irinotecan-based chemotherapies, anti-VEGF and -EGFR agents [1].

This approval was granted based on results of the pivotal phase III RECOURSE trial [2, 3]. Patients with pre-treated mCRC (limited to Eastern Cooperative Oncology Group performance status (ECOG PS) 0 or 1) received either FTD / TPI (*n* = 534) or placebo (*n* = 266) plus best supportive care (BSC). By adding FTD / TPI to BSC, median overall survival (mOS; 7.2 vs 5.2 months; hazard ratio (HR) 0.69, *p* < 0.0001) and median progression-free survival (mPFS; 2.0 vs 1.7 months; HR 0.48, *p* < 0.001) were significantly improved. The toxicity profile was characterised by haematological side effects with neutropenia (38%) and leukopenia (21%) being most frequent. Febrile neutropenia was observed in 4% of patients receiving FTD / TPI. Effect of FTD / TPI on health-related quality of life (QoL) was not assessed based on patient-reported outcomes. However, time to deterioration of the ECOG PS from 0 / 1 to ≥ 2 was significantly longer in patients treated with FTD / TPI (5.7 vs 4.0 months; HR 0.66, *p* < 0.001).

In a *post-hoc* exploratory analysis, the effect of prognostic factors on RECOURSE efficacy outcomes was examined [4]. To this end, 3 subgroups were defined: best, good and poor prognostic characteristics (*N* = 800; BPC, *n* = 153; GPC, *n* = 386; PPC, *n* = 414). Patients with < 3 metastatic sites at inclusion and/or ≥ 18 months from diagnosis to inclusion were considered to have GPC. GPC patients without liver metastasis at inclusion were considered to have BPC. All remaining patients were considered to have PPC. Here, mOS was longer in patients with BPC and GPC compared to patients with PPC (16.4 vs 9.3 vs 5.3 months).

The phase IV TALLISUR (Trifluridine / tipirAcil quaLity of LIfe StUdy in mCRC patients) was a prospective, interventional, multi-centre, Germany-wide, open-label and non-randomised study (EudraCT-Number 2017–000292-83, first registration 19/06/2017). It was designed to generate post-authorisation efficacy and safety data and to assess QoL under FTD / TPI treatment. Patients with pre-treated mCRC were briefed by the treating physician to make an informed decision on either receiving FTD / TPI or solely BSC.

Here, we present the final efficacy data as well as patient-reported QoL results of the TALLISUR study stratified according to prognostic factors or subgroups like age and sidedness complementing previously published results of an interim analysis [5].

## Methods

Methods have been described before[5]. Briefly, FTD / TPI was administered orally twice daily on days 1 to 5 and days 8 to 12 of each 28-day cycle as long as a benefit was observed or until unacceptable toxicity occurred. Based on body surface area, the dose was 35 mg/m^2^ given twice daily and was not allowed to exceed 80 mg per dose. Depending on individual safety and tolerability, a maximum of three dose reduction levels (30, 25 and 20 mg/m^2^) were permitted. In the event of toxicities, the dose interruption, resumption and reduction criteria as stated in the SmPC were followed.

Validated and widely accepted questionnaires were employed to assess HRQoL: European Organisation for Research and Treatment of Cancer (EORTC) QLQ-C30 Version 3.0 [6–8] and EQ-5D-5L [9].

The primary endpoint was pre-defined as the rate of responders with stabilised (> -10 and < 10 scores) or improved (≥ 10 scores) QoL response (QoL-RR). Response was calculated as the mean score of the EORTC QLQ-C30 global health status / QoL scale from the second cycle until the end of treatment / observation compared to the baseline score.

Questionnaires were scheduled to be filled in within 2 days before or on the first day of any treatment / observation cycle. In order to obtain additional information, an extended time period was analysed including questionnaires being filled in between the day after the last administration (FTD / TPI-group) / day 12 (BSC-group) of the previous cycle and the first day of the respective cycle.

Based on baseline characteristics, 3 subgroups were defined according to Supplementary Table 1 as previously described by Tabernero and colleagues [4]. Patients with good prognostic characteristics (GPC) were defined as patients with low metastatic burden (1 or 2 metastatic sites at randomization) and less indolent disease (≥ 18 months from diagnosis of first metastasis). Patients with GPC without liver metastases were defined as patients with best prognostic characteristics (BPC) Patients with poor prognostic characteristics (PPC) were defined as patients with high metastatic burden (≥ 3 metastatic sites at randomization) and/or aggressive disease (< 18 months from diagnosis of first metastasis).

Tumour response was assessed by imaging procedures (magnetic resonance imaging or computed tomography scan) and evaluated according to Response Evaluation Criteria in Solid Tumours (RECIST) version 1.1 [10].

## Results

### Patient enrolment and demographics

**Fig. 1:**
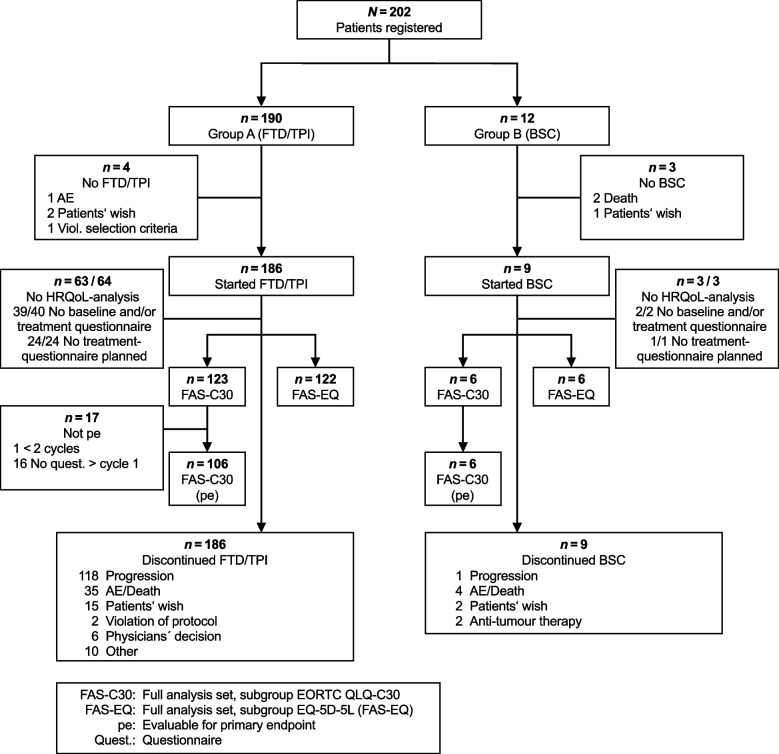
Trial profile

Between 22 September 2017 (first patient included) and 08 January 2019 (last patient included), 195 patients entered the trial across 44 sites in Germany (Fig. 1).

At the time of the interim analysis, 1 patient was wrongly documented to not have received therapy [5]. This patient was now included in this final analysis. Except for one study centre, each centre included at least 1 patient who received at least one FTD / TPI treatment. One centre included only one patient for BSC treatment who did not start close observation in the end.

Due to freedom of choice of each patient to receive active therapy or BSC, the two study arms were imbalanced with respect to the number of patients (186 in the FTD / TPI-group vs 9 in the BSC-group). Therefore, it was not possible to conduct any statistically meaningful analyses using data obtained from patients receiving BSC.

The median duration of FTD / TPI treatment was 68 days (range 1 – 719 days) and the median number of cycles was 3 (range 1 – 23 cycles). 2 patients (1.1%) received a maximum of 23 cycles. The median cumulative FTD / TPI dose administered was 657.4 mg/m^2^ (range 32.5 – 743.9 mg/m^2^) and the median relative dose intensity was 98.1% (range: 4.7 – 110.0%). Throughout the study, 72 patients (38.7%) had at least one cycle with a dose reduction to < 90% of the target dose. During the treatment period, 12 patients (6.5%) received at least one dose of granulocyte colony-stimulating factor.

Baseline characteristics and demographics are shown in Table 1.

**Table 1:** Patient baseline characteristics and demographics

	**FTD/TPI N = 186**	**BSC N = 9**
Age		
median	67.0	78.0
range	40.0–88.0	54.0–82.0
Sex**, *n***(%)		
male	117 (62.9)	5 (55.6)
female	69 (37.1)	4 (44.4)
ECOG performance status**, n**(%)		
0	72 (38.7)	0 (0.0)
1	94 (50.5)	4 (44.4)
2	18 (9.7)	3 (33.3)
3	0 (0.0)	1 (11.1)
unknown	2 (1.1)	1 (11.1)
Primary tumour site		
colon	101 (54.3)	4 (44.4)
rectum	75 (40.3)	3 (33.3)
colon, rectum	10 (5.4)	2 (22.2)
Sidedness of primary tumour		
left	138 (74.2)	6 (66.7)
right	42 (22.6)	2 (22.2)
Both sides	6 (3.2)	1 (11.1)
Metastatic sites (multiple answers)		
liver	150 (80.6)	8 (88.9)
lung	134 (72.0)	5 (55.6)
lymph node	95 (51.1)	5 (55.6)
peritoneum	40 (21.5)	2 (22.2)
other	50 (26.9)	1 (11.1)
Number of metastatic sites, ***n***(%)		
1	23 (12.4)	1 (11.1)
2	72 (38.7)	4 (44.4)
3	65 (34.9)	4 (44.4)
4	23 (12.4)	0 (0.0)
5	3 (1.6)	0 (0.0)
Metastatic manifestation, ***n***(%)		
synchronous metastasis	102 (54.8)	4 (44.4)
metachronous metastasis	63 (33.9)	3 (33.3)
unknown	21 (11.3)	2 (22.2)
Grading (World Health Organization), ***n***(%)		
G1	4 (2.2)	0 (0.0)
G2	128 (68.8)	7 (77.8)
G2 – G3	4 (2.2)	0 (0.0)
G3	27 (14.5)	1 (11.1)
G3 – G4	2 (1.1)	0 (0.0)
G4	3 (1.6)	0 (0.0)
GX	7 (3.8)	0 (0.0)
unknown	11 (5.9)	1 (11.1)
RAS		
wild-type	73 (39.2)	4 (44.4)
mutant	102 (54.8)	4 (44.4)
unknown	11 (5.9)	1 (11.1)
BRAF V600		
wild-type	65 (34.9)	1 (11.1)
mutant	1 (0.5)	0 (0.0)
unknown	120 (64.5)	8 (88.9)
Surgery of primary tumour, ***n***(%)		
no	29 (15.6)	2 (22.2)
yes	157 (84.4)	7 (77.8)
Surgery of metastases, ***n***(%)		
no	102 (54.8)	4 (44.4)
yes	84 (45.2)	5 (55.6)
Radiation**, *n***(%)		
no	134 (72.0)	5 (55.6)
yes	52 (28.0)	4 (44.4)
Number of previous therapy lines for the treatment of mCRC**, *n***(%))		
0	8 (4.3)	2 (22.2)
1	23 (12.4)	1 (11.1)
2	71 (38.2)	3 (33.3)
3	49 (26.3)	0 (0.0)
≥ 4	35 (18.8)	3 (33.3)
Substances of previous systemic anti-CRC therapies, ***n***(%)		
fluoropyrimidine	186 (100.0)	9 (100.0)
irinotecan	172 (92.5)	5 (55.6)
oxaliplatin	175 (94.1)	7 (77.8)
bevacizumab	154 (82.8)	4 (44.4)
anti-EGFR antibodies	73 (39.2)	3 (33.3)
other than the above	48 (25.8)	1 (11.1)

Patients in the FTD / TPI and BSC-group were median 67 (range 40 – 88) or 78 (range 54 – 82) years old and 62.9% or 55.6% male, respectively. 89.2% of patients in the FTD / TPI-group and 44.4% of patients in the BSC-group presented an ECOG PS 0 – 1. Primary tumours were located more often on the left than on the right side of the colon (74.2% vs 22.6% in the FTD / TPI- and 66.7% vs 22.2% in the BSC-group). While most patients were characterised by multiple metastatic sites, 23 patients presented a single metastatic site (15 patients with liver only metastasis, 6 patients with lung only metastasis). 52.6% and 68.4% of patients with ≤ 2 metastatic sites also had a lung or liver metastasis, respectively, compared to 92.3% and 93.4% of patients with > 2 metastatic sites. Most patients received FTD / TPI as a third (38.2%) or fourth (26.3%) line therapy option. Nevertheless, 31 (16.7%) patients received FTD /TPI as first or as second line therapy. The baseline characteristics of these patients are described in more detail in Supplementary Table 2. After end of treatment with FTD / TPI, 36% of patients received subsequent therapy.

## Quality of life

Return rates for EORTC QLC-C30 and EQ-5D-5L are shown in Supplementary Table 3.

Questionnaires from 106 patients receiving FTD / TPI were evaluable for the primary endpoint, QoL-RR. The primary endpoint was reached for FTD / TPI-treated patients with 58.5% QoL-RR (95% CI 48.5 – 68.0). When allowing for the above-mentioned extended period to fill in the questionnaires, QoL-RR was reported even higher with 64.2% (95% CI 54.3 – 73.2).

**Fig. 2:**
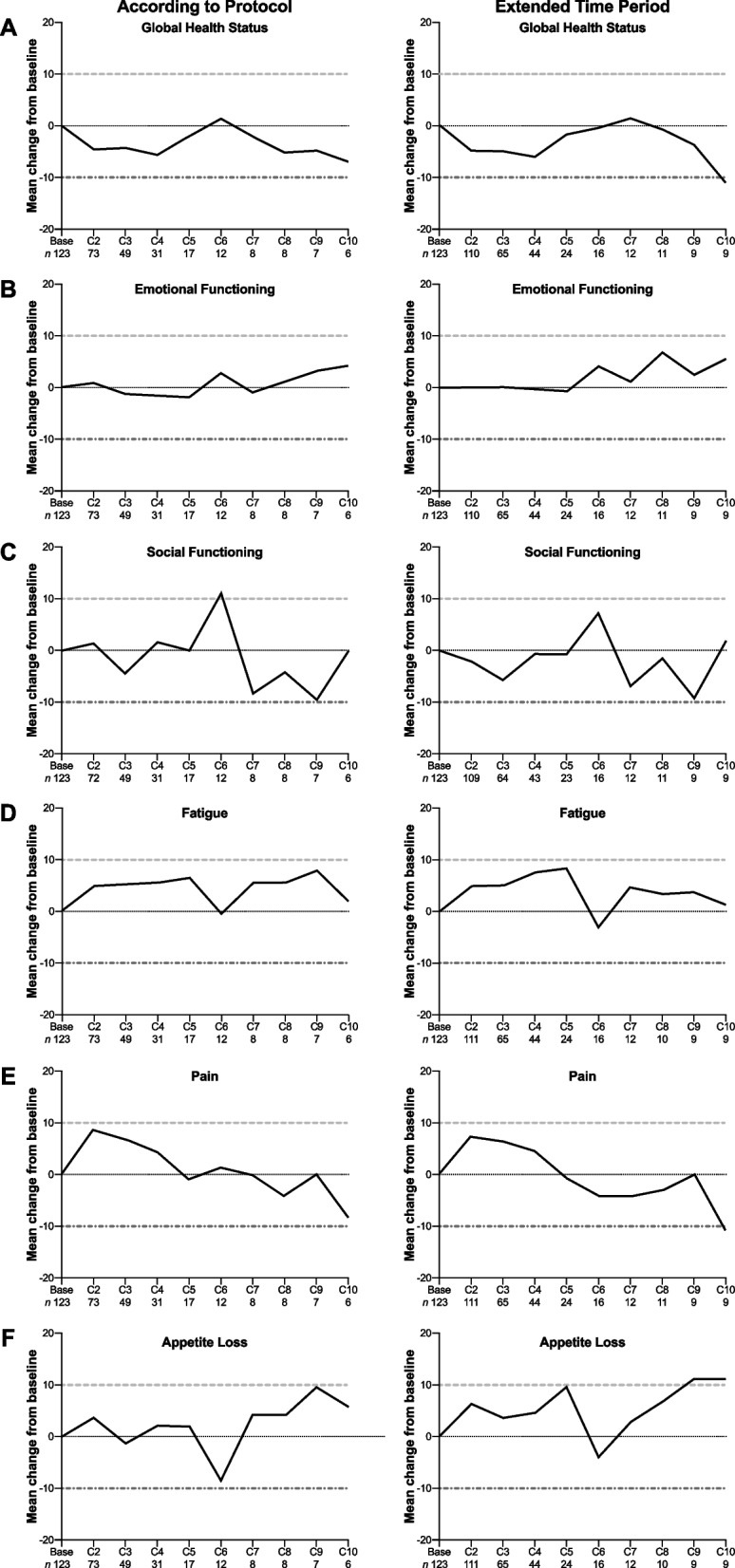
Mean EORTC QLQ-C30 change from baseline

Mean score changes from baseline of selected EORTC QLQ-C30 items up to cycle 6 are shown in Fig. 2 illustrating a maintained QoL during FTD / TPI treatment.

The estimated median time to deterioration of the EORTC QLQ-C30 global health status / QoL score by at least 10 scores was 121 days (95% CI 84.0 – 172.0). Similarly, the estimated median time to deterioration of the EQ VAS was 113 days (95% CI 85.0 – 140.0).

Median time to deterioration of ECOG PS from 0 or 1 to ≥ 2 was 6.2 months (*n* = 166, 95% CI 5.3 – 7.2).

## Efficacy

147 patients (79.0%) in the FTD / TPI group had deceased during the study. Patients without an event were censored at the last contact. Treatment with FTD / TPI was associated with a mOS of 6.9 months (95% CI 6.1 – 8.3) (Supplementary Fig. 1, Supplementary Table 4). 158 events of progression were reported in FTD / TPI-treated patients. For most patients treated with FTD / TPI, progression according to RECIST was the documented PFS event (122 patients, 65.6%), followed by clinical progression (36 patients, 19.4%) and death (19 patients, 10.2%). Median time to progression was 2.5 months (95% CI 2.1 – 3.1) (Supplementary Fig. 2, Supplementary Table 4).

Efficacy data obtained from patients receiving BSC is summarised in Supplementary Table 5.

Response rates of patients treated with FTD / TPI are shown in Supplementary Table 6. Objective response rate (ORR) was 2.2% and disease control rate (DCR) was 27.4%.

## Subgroup analyses

Regarding the primary endpoint, QoL-RR, subgroup analyses are shown in Table 2.

**Table 2:** Quality of life by subgroups

		***N* in group**	**Response achieved**		**95%-CI**	
			***n***	**%**	**Lower limit**	**Upper limit**
Age	< 65 years	40	25	62.5	45.8	77.3
	≥ 65 years	66	37	56.1	43.3	68.3
Sex	male	64	34	53.1	40.2	65.7
	female	42	28	66.7	50.5	80.4
ECOG	0	43	24	55.8	39.9	70.9
	1	54	33	61.1	46.9	74.1
	0—1	97	57	58.8	48.3	68.7
	2 -3	8	4	50.0	15.7	84.3
Localisation of primary tumour	left side	77	42	54.5	42.8	65.9
	right side	24	15	62.5	40.6	81.2
Number of metastatic sites	1	14	7	50.0	23.0	77.0
	> 1	92	55	59.8	49.0	69.9
	1—2	52	30	57.7	43.2	71.3
	> 2	54	32	59.3	45.0	72.4
Duration of metastatic disease	< 18 months	29	18	62.1	42.3	79.3
	≥ 18 months	77	44	57.1	45.4	68.4
Type of metastases	synchronous	53	30	56.6	42.3	70.2
	metachronous	37	22	59.5	42.1	75.2
Number of previous therapy lines of mCRC	0	6	4	66.7	22.3	95.7
	1—2	54	29	53.7	39.6	67.4
	> 2	46	29	63.0	47.5	76.8
	0—2	60	33	55.0	41.6	67.9
	2	42	23	54.8	38.7	70.2
Previous therapies of (m)CRC—all substances given	no	80	46	57.5	45.9	68.5
	yes	26	16	61.5	40.6	79.8
Prognostic groups	best prognostic group	9	5	55.6	21.2	86.3
	good prognostic group	30	16	53.3	34.3	71.7
	poor prognostic group	67	41	61.2	48.5	72.9

Results were comparable between patients with BPC, GPC and PPC. Also, patients with right- and left-sided primary tumour had a comparable QoL-RR. Similarly, QoL-RR of patients with synchronous and metachronous metastases did not differ greatly. Also, patients who had been diagnosed < 18 compared to ≥ 18 months ago had similar QoL-RR. Furthermore, neither the number of metastatic sites nor the number of previous lines of therapies affected QoL-RR. Patients with ECOG PS ≥ 2 presented a comparable QoL-RR compared to patients with ECOG PS < 2. Interestingly, however, male patients had a slightly lower QoL-RR compared to female patients.

Regarding OS, a univariate Cox regression analysis was performed based on patients’ baseline characteristics (Supplementary Table 7). Two characteristics showed statistical significance: ECOG 0 – 1 compared to ECOG 2 – 3 and diagnosis of metastatic disease < 18 compared to ≥ 18 months ago. However, according to a multivariate Cox regression analysis, no statistical significance was observed (Supplementary Table 8).

**Fig. 3:**
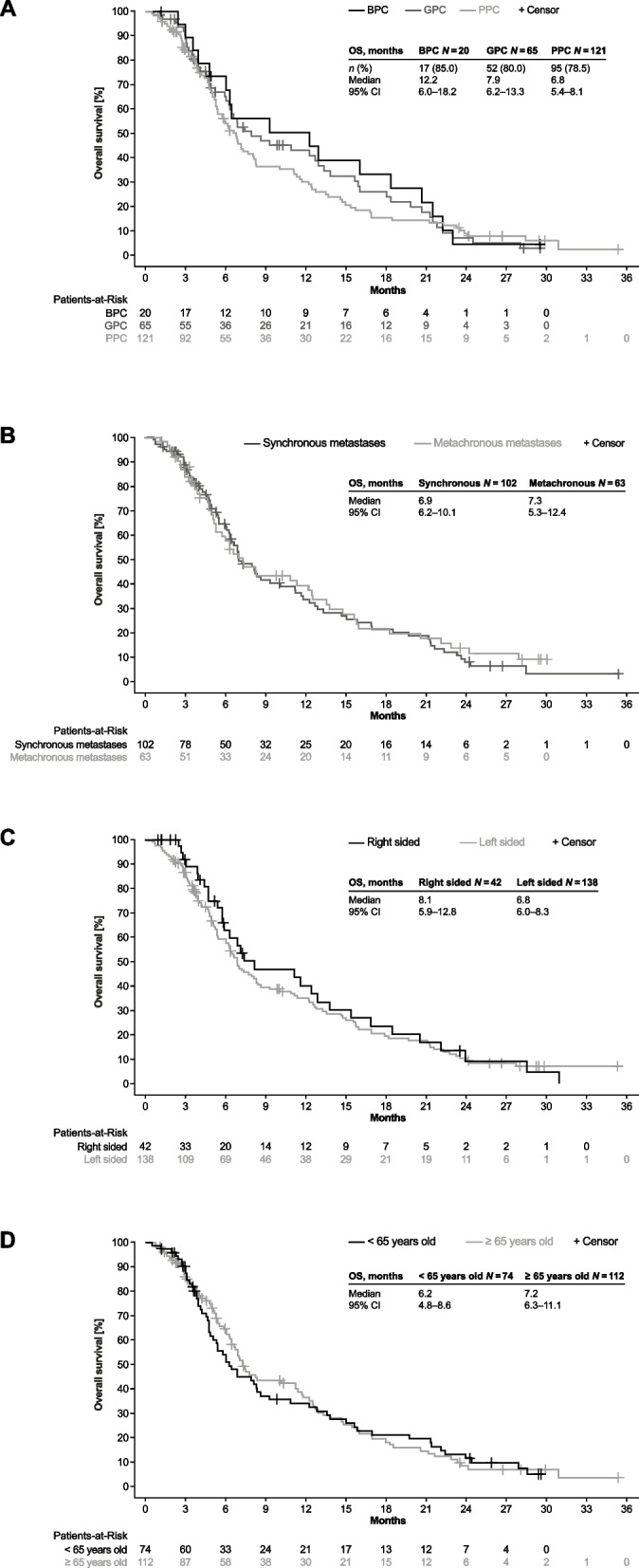
Overall survival by subgroups

In the subgroups of patients with BPC (15.9 months [95% CI 6.3 – 21.4]) or GPC (9.2 months [95% CI 6.5 – 13.8]), mOS was longer as compared to PPC (6.8 months [95% CI 5.7–10.1]) (Fig. 3a).

Survival was comparable between patients with synchronous and metachronous metastases (Fig. 3b), patients with right and left sided primary tumour (Fig. 3c) and between younger and elderly patients (Fig. 3d). Also, similar OS was observed between patients who received FTD / TPI as first line therapy (mOS 6.0 months, *n* = 8) or after 1 or 2 previous lines of therapy (mOS 6.5 months, *n* = 94). The median duration of treatment for each subgroup is shown in Supplementary Table 9.

## Safety

At least one treatment emergent adverse event (TEAE) of any grade occurred in 179 FTD / TPI-treated patients (96.2%) and in 7 patients receiving BSC (77.8%) (Supplementary Table 10). At least one TEAE of grade ≥ 3 was experienced by 125 FTD / TPI-treated patients (67.2%) and by 6 patients receiving BSC (66.7%). Serious TEAEs were reported for 83 FTD / TPI-treated patients (44.6%) and for 5 patients receiving BSC (55.6%). Serious TEAEs of grade 5 that resulted in death occurred in 25 FTD / TPI-treated patients (13.4%), of which 13 (7.0%) were related to cancer progression, and in 1 patient receiving BSC (11.1%). Of the 185 FTD / TPI-treated patients, 53 (28.5%) discontinued study treatment, 23 (12.4%) experienced a dose reduction and 55 (29.6%) a dose delay due to a TEAE. The most frequently reported TEAEs of any grade for FTD / TPI-treated patients were neutropenia (29.6%), anaemia (24.7%), nausea (23.7%), fatigue (23.1%), diarrhoea (21.5%), leukopenia (19.9%), decreased appetite (15.1%) and vomiting (15.1%). Febrile neutropenia of grade 3 occurred in 2 patients treated with FTD / TPI (1.1%), but in none receiving BSC.

## Discussion

The aim of the TALLISUR study was to complement the pivotal phase III RECOURSE trial with post-authorisation efficacy and safety data and patient-reported outcomes on QoL from German patients [2, 3].

Here, we reported the final efficacy, safety and QoL results which were in line with results from an interim analysis published before [5]. Additionally, we presented the survival and QoL results of comprehensive subgroup analyses.

In a *post-hoc* exploratory analysis of the RECOURSE trial, the effect of prognostic factors were investigated and 3 subgroups were defined: BPC, GPC, PPC [4]. Low metastatic burden (≤ 2 metastatic sites at inclusion) and indolent disease (≥ 18 months from diagnosis of metastatic disease to inclusion) were factors of good prognosis with regard to OS and PFS. We applied these criteria for the population of the TALLISUR trial which more accurately reflects daily clinical practice (older and irrespective of ECOG PS). Independent of other baseline characteristics such as ECOG PS and age, low metastatic burden and indolent disease were factors of good prognosis for survival of patients within the TALLISUR trial. mOS for BPC, GPC and PPC was 15.9, 9.2 and 6.8 months, respectively. This was comparable to results of the RECOURSE trial (16.4, 9.3 and 5.3 months, respectively). The same analysis has been performed on 300 German patients treated in daily clinical practice [11]. In line with results of TALLISUR, patients with BPC presented a longer survival (BPC *n* = 54 vs GPC *n* = 147 vs PPC *n* = 96: 16.2 vs 9.8 vs 6.3 months; 95% CI 9.7 – 19.4 vs 8.6 – 11.7 vs 4.5 – 7.8).

Interestingly, QoL-RR was comparable between all three subgroups. This observation is particularly important, since QoL-RR is the major objective when applying last line treatment. The reason for this observation remains unclear, however. Although the RECOURSE trial reported a longer mPFS and mOS for patients treated in fourth and fifth line compared to third line, no difference was observed in regards of the number of metastatic sites (1 – 2 vs ≥ 3). However, QoL was not assessed in RECOURSE at all. Whereas patients with BPC profited the most, patients from all prognostic subgroups evidently benefitted from treatment with FTD / TPI. Time of onset of metastasis (synchronous vs metachronous), localisation of primary tumour (right vs left) and age at inclusion (≥ 65) did not affect efficacy of FTD / TPI nor its effect on QoL.

Male patients had a slightly lower QoL-RR compared to female patients. This could also be explained by male patients generally being older than female patients (62.5% of patients ≥ 65 years old were male) when enrolling into the study.

When interpreting the QoL results, one must bear in mind that the return rates of questionnaires were higher when considering the extended period (Supplementary Table 3). This also illustrates that especially with late-stage cancer patients, formal assessment of patient-reported outcomes is typically an arduous task.

TEAEs observed in TALLISUR were in line with the safety profile of FTD / TPI [2, 3]. This is particularly relevant for TEAEs believed to likely affect QoL, such as nausea, vomiting, diarrhoea, and fatigue [12].

As indicated by these subgroup analyses, FTD / TPI is a viable option for virtually all patients who are not considered candidates for other available therapies. This is also stressed by 16.7% of patients receiving FTD / TPI as first or as second line therapy.

The TALLISUR results are in line with previous research investigating the effect of FTD / TPI on QoL [13–15]. While stabilising disease, FTD / TPI is also associated with maintaining QoL, two crucial objectives in the late stage setting of mCRC [16, 17].

Recently, FTD / TPI has been approved by EMA in combination with bevacizumab for the treatment of mCRC patients after two prior lines of therapy based on the pivotal phase III SUNLIGHT trial [18]. In line with the TALLISUR results, QoL was maintained for patients in the monotherapy arm as well as for patients in the combination arm [19].

Based on the FRESCO-2 trial, fruquintinib has been approved by EMA for treatment of mCRC patients who have been previously treated with available standard therapies and who have progressed on or are intolerant to treatment with either FTD / TPI or regorafenib [20]. Here, patients who had been treated with FTD / TPI and/or regorafenib were randomised to receive either fruquintinib or placebo. This situation does not reflect the situation of the TALLISUR study, however. Beyond therapy with FTD / TPI monotherapy or regorafenib monotherapy, QoL was not negatively impacted by fruquintinib therapy [21].

## Conclusions

In summary, the TALLISUR trial provided patient-reported QoL and post-authorisation efficacy and safety data from German patients with pre-treated mCRC receiving FTD / TPI. Overall, treatment with FTD / TPI prolonged survival and delayed progression with a manageable toxicity profile while concomitantly being associated with maintained QoL.

### Supplementary Information

Supplementary Material 1: Supplementary Table 1 Definition of subgroups.

Supplementary Material 2: Supplementary Table 2 Baseline characteristics of patients treated with FTD/TPI according to number of previous therapy lines for the treatment of mCRC.

Supplementary Material 3: Supplementary Table 3 Return rates of questionnaires.

Supplementary Material 4: Supplementary Table 4 Time to event (days) in patients receiving FTD/TPI.

Supplementary Material 5: Supplementary Table 5 Time to event (days) in patients receiving BSC.

Supplementary Material 6: Supplementary Table 6 Response rates.

Supplementary Material 7: Supplementary Table 7 Overall survival—univariate Cox regression analysis.

Supplementary Material 8: Supplementary Table 8 Overall survival—multivariate Cox regression analysis.

Supplementary Material 9: Supplementary Table 9 Duration of treatment by subgroups. *Duration of treatment known for 183/186.*

Supplementary Material 10: Supplementary Table 10 Treatment-emergent adverse events.

Supplementary Material 11: Supplementary Fig. 1 Overall survival.

Supplementary Material 12: Supplementary Fig. 2 Progression-free survival.

## Data Availability

The datasets used and/or analysed during the current study are available from the corresponding author on reasonable request.
