# Supplementary figures and images for: Subgroup analyses from patients with pre-treated metastatic colorectal cancer receiving trifluridine/tipiracil: results of the TALLISUR trial
Source: BMC Cancer. 2024 Jul 23;24:887. doi: 10.1186/s12885-024-12599-7 (PMC11267741; doi:10.1186/s12885-024-12599-7)

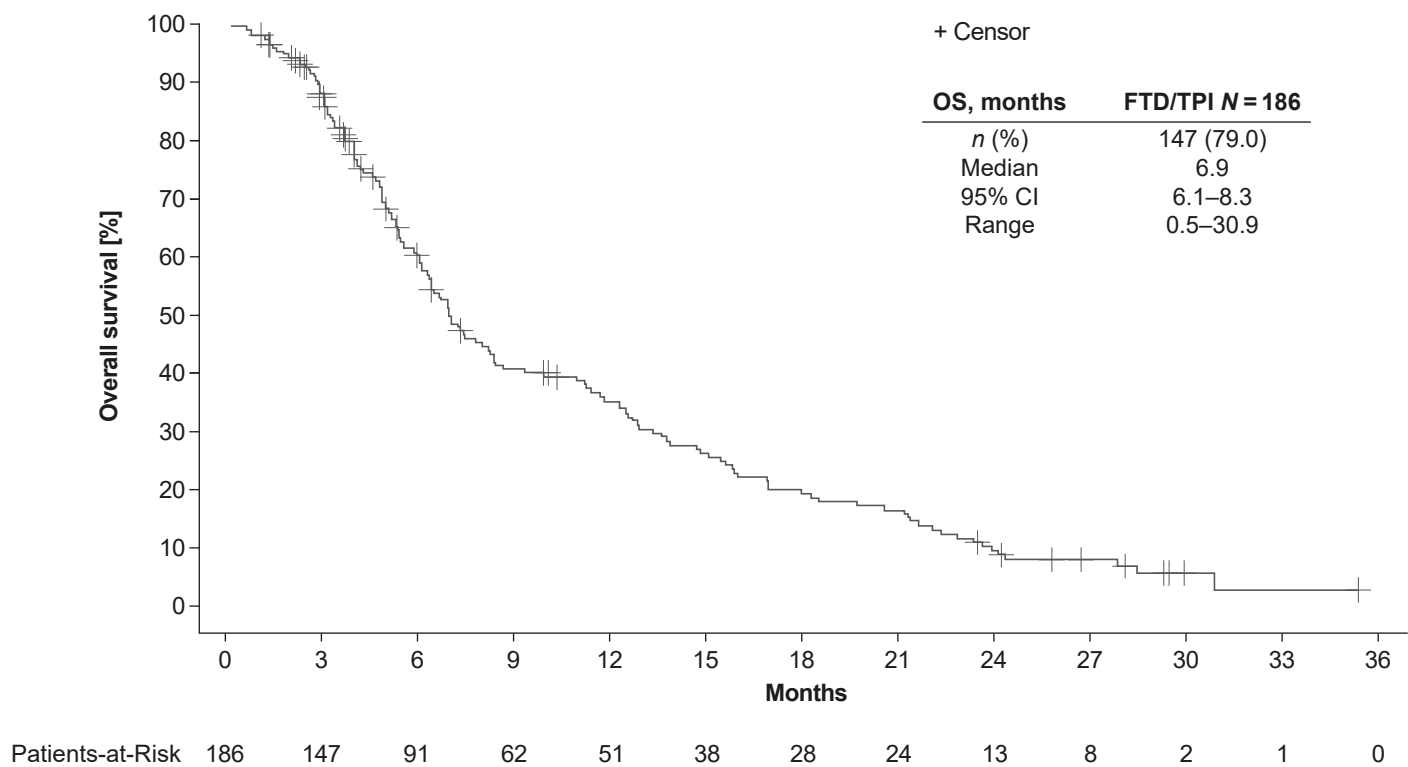

Supplementary Figure 1 Overall survival

Supplement: Supplementary file 11 — Supplementary Material 11: Supplementary Fig. 1 Overall survival. [file 12885_2024_12599_MOESM11_ESM.pdf]

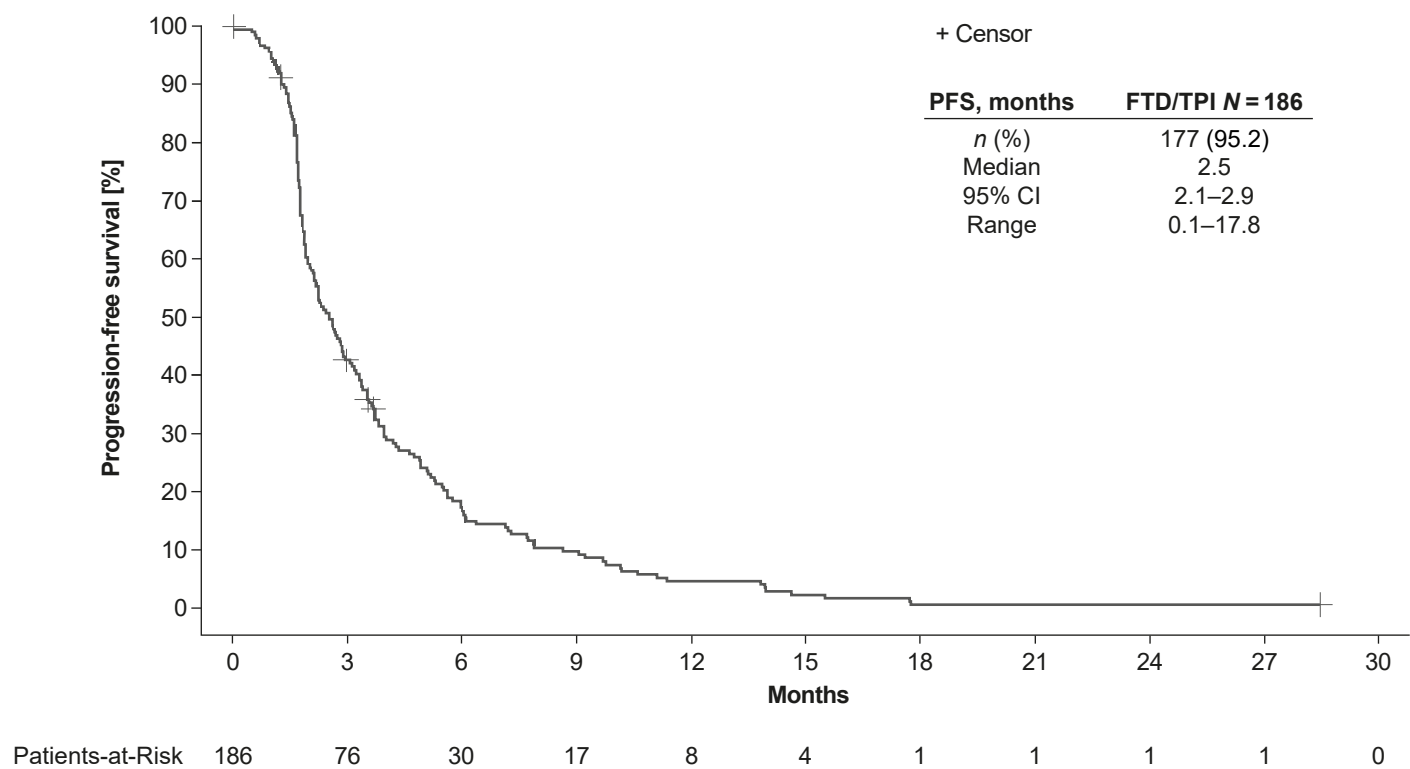

Supplementary Figure 2 Progression-free survival

Supplement: Supplementary file 12 — Supplementary Material 12: Supplementary Fig. 2 Progression-free survival. [file 12885_2024_12599_MOESM12_ESM.pdf]
